# Binge Alcohol Exposure Transiently Changes the Endocannabinoid System: A Potential Target to Prevent Alcohol-Induced Neurodegeneration

**DOI:** 10.3390/brainsci7120158

**Published:** 2017-11-29

**Authors:** Daniel J. Liput, James R. Pauly, Audra L. Stinchcomb, Kimberly Nixon

**Affiliations:** Department of Pharmaceutical Sciences, College of Pharmacy, University of Kentucky, Lexington, KY 40536, USA; daniel.liput@nih.gov (D.J.L.); jim.pauly@uky.edu (J.R.P.); Astinchc@rx.umaryland.edu (A.L.S.)

**Keywords:** alcohol, alcoholism, CB1, ethanol, endocannabinoid, N-acylethanolamine, FAAH, neurodegeneration

## Abstract

Excessive alcohol consumption leads to neurodegeneration, which contributes to cognitive decline that is associated with alcohol use disorders (AUDs). The endocannabinoid system has been implicated in the development of AUDs, but little is known about how the neurotoxic effects of alcohol impact the endocannabinoid system. Therefore, the current study investigated the effects of neurotoxic, binge-like alcohol exposure on components of the endocannabinoid system and related N-acylethanolamines (NAEs), and then evaluated the efficacy of fatty acid amide hydrolase (FAAH) inhibition on attenuating alcohol-induced neurodegeneration. Male rats were administered alcohol according to a binge model, which resulted in a transient decrease in [^3^H]-CP-55,940 binding in the entorhinal cortex and hippocampus following two days, but not four days, of treatment. Furthermore, binge alcohol treatment did not change the tissue content of the three NAEs quantified, including the endocannabinoid and anandamide. In a separate study, the FAAH inhibitor, URB597 was administered to rats during alcohol treatment and neuroprotection was assessed by FluoroJade B (FJB) staining. The administration of URB597 during binge treatment did not significantly reduce FJB+ cells in the entorhinal cortex or hippocampus, however, a follow up “target engagement” study found that NAE augmentation by URB597 was impaired in alcohol intoxicated rats. Thus, potential alcohol induced alterations in URB597 pharmacodynamics may have contributed to the lack of neuroprotection by FAAH inhibition.

## 1. Introduction

Excessive alcohol consumption represents a significant social, economic, and public health problem in the United States, with recent estimates showing 13.9% of Americans meeting the diagnostic criteria for an alcohol use disorder (AUD) [1]. Importantly, up to 76% of heavy drinkers consume alcohol in a binge manner [2], which is especially detrimental to brain physiology and structure, resulting in an increased risk for alcohol dependence, co-morbid psychiatric disorders, and alcoholic neuropathology [3,4,5,6]. The negative impact of excessive alcohol consumption on health and society warrants identification of new drug targets and development of pharmacotherapies for the treatment of AUDs. 

Current Food and Drug Administration (FDA) approved pharmacotherapies have had limited clinical utility due to their modest efficacy and/or compliance [7,8]. The lack of viable treatment options is highlighted by the fact that less than 15% of patients with an AUD are prescribed medication [9,10]. AUDs are heterogeneous in nature and it is unlikely that a single drug or single drug target will be effective for all patients [8]. Therefore, current medication development for AUDs needs to focus on increasing the repertoire of available pharmacotherapies to increase effective treatment options across the spectrum of the disorder. To that end, the identification of new drug targets that underlie AUDs, such as alcohol-induced neurodegeneration, should aid in medication development efforts and improve clinical outcomes. 

Neurodegeneration, including reductions in cortical gray matter and white matter volume, is commonly observed following long-term alcohol consumption [11,12]. Alcohol is particularly damaging to the frontal and temporal lobes [13,14] and the hippocampus [15,16,17], which may explain, in part, deficits in executive function, learning and memory, and emotional processing in alcoholics [18,19]. Compromised structural integrity and cognitive function may contribute to the chronic and relapsing nature of alcoholism [20,21], therefore, it is hypothesized that neuroprotective agents may reduce alcohol-induced neurodegeneration, restore cognitive function, and improve treatment outcomes for alcoholism. 

The endocannabinoid (eCB) system has received considerable attention in the addiction field over the last decade because genetic variants in cannabinoid genes are associated with a high risk for drug dependence and this system is profoundly dysregulated by these substances, including alcohol. For example, single nucleotide polymorphisms in *CNR1* (cannabinoid receptor 1) [22,23] and *FAAH* (fatty acid amide hydrolase) [24,25], the catabolic enzyme for n-acylethanolamides (NAEs), including anandamide (AEA), are associated with alcohol dependence and studies have found widespread cannabinoid receptor 1 (CB1) downregulation in the central nervous system (CNS) of human alcoholics [26], an effect that is recapitulated in rodent models. In fact, studies using a variety of experimental models have shown alcohol-induced alterations on nearly every component of the eCB system [27,28] and pharmacological modulation of the eCB system impacts alcohol consumption and preference [29]. Although many preclinical studies have examined the role of eCBs on the different facets of alcohol dependence, few have studied this system in models of neurotoxic, binge-like exposure models, which may have important implications for the development of neuroprotective therapeutics for alcoholism.

The eCB system has emerged as a potent neuroprotective target in a variety of models of neurodegeneration [30]. Several studies have observed elevated eCB tissue content following acute neuronal injury and during chronic neuroinflammation [31,32,33,34,35,36,37,38]; and, CB1 null-mutant mice are more susceptible to pathological insults [35,39]. Furthermore, cannabinoid receptor agonists are neuroprotective in many experimental models of neurodegeneration [40,41]. The primary mechanisms by which cannabinoid agents afford neuroprotection, include attenuation of excitotoxicity, neuroinflammation, and oxidative stress [30,42], all of which are implicated in models of AUDs [43]. Therefore, the eCB system may be a viable target to prevent alcohol-induced neurodegeneration. 

Much of the current work on eCBs has utilized agonists for CB1 and cannabinoid receptor 2 (CB2) to demonstrate neuroprotection, but the use of these agonists in a clinical setting may be limited. CB1 agonists are associated with untoward psychotropic effects and abuse liability, which likely outweigh the benefits of these agents [30,44]. Further, although CB2 agonists are not psychoactive and have neuroprotective properties [45], the role of CB2 receptors in the CNS is still unclear. Alternatively, indirect modulation of the eCB system by inhibiting the catabolism of eCBs may prove more advantageous for the treatment of neurodegeneration. Inhibition of FAAH recapitulates a distinct subset of CB1-dependent effects, such that the beneficial effects of the eCB system may be exploited, while the untoward effects avoided. For example, FAAH inhibitors afford neuroprotection in models of kainic acid-induced excitotoxic brain damage and following focal cerebral ischemia [32,46,47,48], while being devoid of psychoactive properties [49]. Additionally, FAAH inhibitors are dependent on “on-demand” synthesis of eCBs and, as such, their activity is hypothesized to be greater in degenerative tissue [50] where elevations in eCBs and NAEs are commonly observed [31,34,36]. 

To date, little data exists addressing the neuroprotective effects of the eCB system in models of alcohol-induced neurodegeneration. One report found that targeting the eCB system is neuroprotective in an in vitro model of alcohol withdrawal [51], however excitotoxicity that is associated with withdrawal is not the sole mediator of alcohol-induced neurodegeneration [43]. In fact, alcohol neurotoxicity is observed in experimental models in the absence of an overt withdrawal syndrome [52,53,54,55]. In another study, FAAH inhibition attenuated oxidative stress in a binge model of alcohol intake, but this study did not examine neurodegeneration [56]. To date, no studies have examined the neuroprotective properties of the eCB system in an in vivo model of alcohol-induced neurodegeneration. Therefore, the current study characterized the effect of binge alcohol exposure on components of the eCB system, and then examined the neuroprotective effects of targeting the eCB system by FAAH inhibition using an established model of alcohol-induced neurodegeneration.

## 2. Materials and Methods

A total of 137 adult male Sprague Dawley rats (~330 g or ~P75 on arrival; Charles River, Raleigh, NC) were used in these studies (five rats were lost to gavage error). All of the procedures followed the *Guide for the Care and Use of Laboratory Animals* [57] and were approved by the University of Kentucky Institutional Animal Care and Use Committee. Rats were single-housed in Plexiglas cages in an AAALAC approved vivarium at the University of Kentucky on a 12 h light/dark cycle with *ad libitum* access to water and rat chow except during binge ethanol treatment, when chow was removed. Rats were acclimated and handled for at least three days prior to initiation of binge treatment, and then randomly assigned to treatment groups. Rats are assigned a subject number so that all experimenters are blind to experimental groups from tissue isolation through collection of data.

### 2.1. Binge Ethanol Treatment

Rats were treated according to a modified Majchrowicz four-day binge model, as previously described [58]. This model was chosen because it maintains intoxicating blood ethanol concentrations (BECs) similar to binge-pattern drinking alcoholics [59,60], the most common, but damaging pattern of intake [2,4], and it results in a well-defined pattern of neurodegeneration in corticolimbic structures [61,62,63,64]. Every 8 h for one (3 ethanol doses), two (6 ethanol doses), or four (12 ethanol doses) days rats were administered either a 25% (*w*/*v*) ethanol diet (Vanilla Ensure Plus^®^; Abbott Laboratories, Columbus, OH, USA) or an isocaloric control diet (dextrose, water and Vanilla Ensure Plus^®^) by intragastric intubation. Ethanol treated rats were administered a 5 g/kg priming dose of ethanol, while subsequent doses were titrated based on the rat’s intoxication state, according to the following scale: 0, normal rat (5 g/kg); 1, hypoactive (4 g/kg); 2, ataxic (3 g/kg); 3, delayed righting reflex and lack of abdominal elevation (2 g/kg); 4, lack of righting reflex with intact eye blink reflex (1 g/kg, 2 mL H_2_O); 5, loss of eye blink reflex (0 g/kg, 2 mL H_2_O). Controls received the average volume of liquid administered to ethanol rats for that session (for detail see [58,65]). BECs were measured in trunk blood, collected during euthanasia (~2 h following the last dose of ethanol) from rats treated for one or two days, or in tail blood collected 90 min following the 7th dose of ethanol. Approximately 150 µL of blood was collected into microcentrifuge tubes containing heparin (5 µL; AAP pharmaceuticals, Schaumberg, IL, USA), centrifuged at 1800× *g* for 5 min, and stored at −20 °C. BECs were determined in triplicate using an AM1 alcohol analyzer (Analox, Lunenburg, MA, USA) calibrated to a 300 mg/dL standard. 

### 2.2. CB1 Receptor Autoradiography

Rats were euthanized after one day (~2 h after dose 3), two days (~2 h after dose 6), or four days (~2 h after dose 12) of binge ethanol treatment by rapid decapitation and whole brains were immediately dissected, flash frozen in isopentane on dry ice, and stored at −80 °C until sectioning. Brains were sectioned at 16 μm in a 1:12 series using a Leica CM1850 cryostat (Leica, Nussloch, Germany) and mounted on Superfrost Plus^®^ slides (Fisher Scientific, Pittsburgh, PA, USA). After sectioning, slides were stored overnight at 4 °C under desiccation and then stored at −80 °C. CB1 receptor autoradiography was performed using the CB1 receptor agonist, [^3^H]-CP-55,940, similar to that previously described (Perkin Elmer, Specific Activity = 173 Ci/mM; [66]). Sections were thawed overnight at 4 °C under desiccation and brought to room temperature before binding. Sections were pre-incubated in Tris-HCl buffer (pH 7.4) containing Tris-HCl (50 mM), NaCl (120 mM), KCl (5 mM), MgCl_2_ (1 mM), CaCl_2_ (2.5 mM), and 5% BSA for 30 min, and then placed in fresh Tris-HCl buffer with the addition of 2.5 nM [^3^H]-CP-55,940 for 2 h at 37 °C. Following incubation with the radioligand, sections were washed in Tris-HCl buffer (pH 7.4) containing 1% BSA and no binding salts at 4 °C for 1 h. Sections were washed again with fresh Tris-HCl buffer containing 1% BSA at 4 °C for 3 h and then in Tris-HCl buffer without BSA at 4 °C for 5 min. Last, sections were washed briefly in 4 °C dH_2_O and dried under gentle air stream overnight. After drying, all of the slides were placed in Fisher Biotech autoradiographic cassettes and slides were exposed to Kodak Biomax film for 27 days prior to film development. 

Films were imaged using a Macintosh-based image analysis system and quantified by a blinded experimenter using ImageJ (version 1.59, NIH, Bethesda MD, USA). Initially, CB1 receptor autoradiography was quantified in the entorhinal cortex (Bregma coordinates −5.40 to −6.48; [67]) and hippocampus (Bregma −2.76 to −4.56), the two brain regions most susceptible to binge ethanol-induced neurotoxicity. In subsequent analysis, subregions of the hippocampus were analyzed separately. The dorsal dentate gyrus, CA1 striatum oriens and CA3 striatum oriens were analyzed between Bregma −2.76 to −4.56, while the ventral dentate gyrus was defined as the lateral striatum oriens between Bregma −5.40 to −6.12 and from the most ventral point to the height of the rhinal fissure.

### 2.3. N-Acylethanolamine Extraction and Quantification

Processing and quantification procedures for AEA, oleoylethanolamine (OEA), and palmitoylethanolamine (PEA) were performed exactly as described previously [68]. Briefly, rats were euthanized by rapid decapitation ~2 h after the last dose of ethanol, hippocampi, and entorhinal cortices were dissected and flash frozen in an ethanol (70%)/dry ice slurry and stored at −80 °C. Experiments for two day and four day time points were performed independently; therefore, tissue samples were collected on separate occasions. Tissue was weighed and homogenized with equal volumes of ice cold saline in a silanized microcentrifuge tube using a Sonic Dismembrator (Fisher Scientific, Fairlawn, NJ, USA). NAEs were extracted from 100 µL of homogenate using 1 mL acetonitrile (ACN). Samples were vortexed for approximately 30 s and centrifuged at 13,000× *g* at 4 °C for 20 min. Supernatants were transferred to a silanized test-tube and evaporated under a gentle N_2_ airstream. NAEs were extracted two additional times and supernatants were pooled by evaporating extracts in the same silanized test-tube. Samples were reconstituted in 100 µL ACN by vortexing and sonication in an ice bath. To remove precipitates, samples were centrifuged at 13,000× *g* at 4 °C. Samples were then transferred to an HPLC vial containing a silanized microinsert and stored in an autosampler at 4 °C. 

High performance liquid chromatography was performed using a Waters Alliance 2695 LC pump (Waters, Milford, MA, USA) equipped with a Waters Alliance 2695 autosampler. Separation was achieved using a Waters Symmetry^®^ C_18_ (2.1 × 150 mm, 5 µm) column coupled with a Waters Symmetry^®^ C_18_ guard column (2.1 × 10 mm, 3.5 µm). A gradient elution protocol was used with mobile phase A consisting of 1 mM ammonium acetate with 0.1% acetic acid (*v*/*v*) in methanol and mobile phase B consisting of 1 mM ammonium acetate with 0.1% acetic acid (*v*/*v*) and 5% methanol in water. Initial conditions were set at 70% A and 30% B. A was increased linearly to 85% over 25 min and maintained for 1 min, increased linearly to 100% over 1 min and held at 100% for 5 min. Finally, A was returned linearly to 70% over 1 min and was held for 10 min for column equilibration. Flow rate was maintained at 0.3 mL/min. A Micromass ZQ^®^ (Waters, Milford, MA, USA) mass spectrometer (MS) with electrospray ionization (ESI) was set to the following: nitrogen desolvation gas 450 L/h, nitrogen cone gas 50 L/h, source temperature 120 °C, desolvation temperature 250 °C, capillary voltage 3.5 kV, cone voltage 25 kV, extractor voltage 5.0 kV, RF lens voltage 0.5 kV. ESI was set to the positive mode and selective ion monitoring was set to the following protonated ions, *m*/*z* 348.28 [M + H]^+^ (AEA), *m*/*z* 326.6 [M + H]^+^ (OEA), and *m*/*z* 300.5 [M + H]^+^ (PEA) with dwell times of 0.3 s for each ion. NAE concentrations were back calculated using standard curves prepared from tissue standards and quality controls were run periodically. 

### 2.4. URB597 Regimen

The FAAH inhibitor, URB597, was dissolved in dimethyl sulfoxide (DMSO) at a concentration of 1.0 mg/mL. For the neuroprotection study, no injection, URB597 (0.3 mg/kg, i.p.) or vehicle was administered twice daily (11:00 a.m. and 11:00 p.m.) starting after the third intubation of ethanol or control diet and continued for the duration of binge treatment. This dosing regimen was chosen based on previous studies demonstrating maximal FAAH inhibition for at least 12 h with 0.3 mg/kg URB597 [69]. In a separate experiment, to confirm FAAH inhibition, no injection, a single injection of vehicle, or a single dose of URB597 (0.3 mg/kg, i.p.) was administered following the third intubation of ethanol or control diet and 2 h prior to euthanasia. This time interval was chosen because URB597 maximally elevates NAEs 2 h following administration [69].

### 2.5. FluoroJade B Staining and Quantification

After four days of binge treatment (12 doses), rats were overdosed with sodium pentobarbital (Fatal Plus^®^, Vortech Pharmaceuticals, Dearborn, MI, USA) then perfused transcardially with 0.1 M phosphate buffed saline (PBS, pH 7.4) followed by 4% paraformaldehyde. Brains were extracted and post-fixed in 4% paraformaldehyde overnight, rinsed, and stored in PBS at 4 °C. Brains were sectioned at 40 μm in a 1:12 series using a Leica vibrating microtome (Leica Microsystems, Wetzlar, Germany) and stored in cyroprotectant at −20 °C. FluoroJade B (FJB; Millipore, Billerica, MA, USA) staining was performed on every 6th section, as described in [55,62]. Profile counts of FJB positive (+) cells were obtained by a blinded experimenter in the entorhinal cortex (Bregma −5.20 to −7.44) and ventral dentate gyrus (Bregma −5.20 to −6.84; [67]) on an Olympus BX-51 microscope equipped for epifluorescence with a 488λ filter cube. FJB+ cells were reported as the number of FJB+ cells/section as the dark background, and therefore the lack of independently definable landmarks coupled with relatively low number of FJB+ profiles makes stereological estimates inappropriate [70]. Strict criteria were used to identify FJB+ cells: cells were included if they were in cortical layers II or III (entorhinal cortex) or in/adjacent to the granular cell layer (dentate gyrus), displayed a pyramidal cell body characteristic of neurons, and/or had observable proximal dendrites. FJB+ cells were rarely observed in control rats (<1 cell/section) regardless of treatment, and were not significantly different; therefore, controls were collapsed into a single control group. 

### 2.6. Statistical Analysis

Statistical analyses were performed using GraphPad Prism (GraphPad version 4.03, La Jolla, CA, USA). All of the values are reported as mean ± SEM and statistical significance was accepted at *p* < 0.05. Intoxication behavior and daily dose were analyzed by Mann Whitney or Kruskal-Wallis test followed by Dunns post-hoc test. BECs, CB1 autoradiography and NAE data were analyzed by analysis-of-variance (ANOVA) followed by planned comparisons using Bonferroni corrected post-hoc t-tests when appropriate. FJB+ cell counts were log transformed and analyzed by ANOVA followed by planned post-hoc t-tests. Pearson correlations were performed to assess the relationship between BECs and NAE concentration for rats treated with binge ethanol and URB597. 

## 3. Results

### 3.1. Binge Ethanol Exposure Transiently Decreases [^3^H]-CP-55,940 Binding but Does Not Alter NAE Tissue Content

CB1 agonist binding and tissue content of three prototypical NAEs (including the CB1 receptor agonist AEA) was measured in rats exposed to a neurotoxic binge pattern of ethanol exposure. Separate cohorts of rats were used for CB1 receptor autoradiography and the measurement of NAE tissue content; therefore, binge treatment parameters were compared between experiments. All of the ethanol treated rats displayed intoxication behaviors that are typical for the binge ethanol exposure paradigm used, ranging from hypoactivity to lack of righting reflex, or in rare cases, the loss of the eye blink reflex [58]. Scored intoxication behaviors, ethanol doses and BECs were statistically indistinguishable between cohorts of rats used for either CB1 receptor autoradiography or NAE quantification at corresponding time points (Table 1). These binge parameters were not compared across durations of exposure as dosing differs across the binge model.

**Figure 1:**
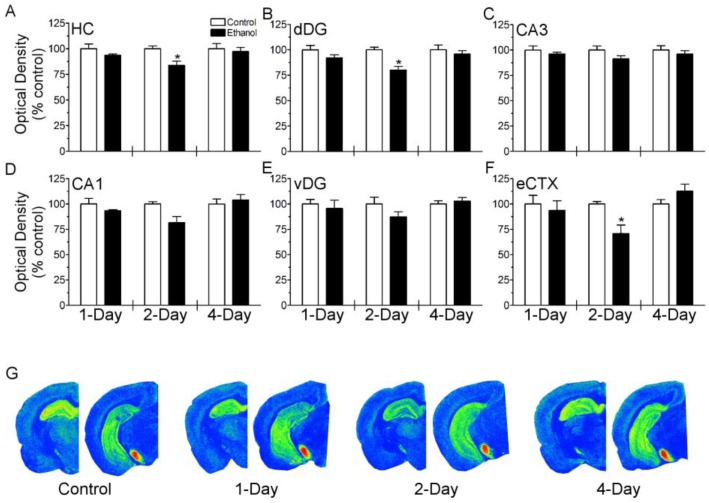
Assessment of CB1 receptor agonist binding in the hippocampus and entorhinal cortex following binge ethanol treatment. Rats (*n* = 5–6/group) were administered ethanol or an isocaloric control diet for 1, 2, or 4 days and [^3^H]-CP-55,940 binding was measured by autoradiography. (A–F) Optical density was quantified in each brain region and expressed as percent of time point matched controls. (G) Representative pseudocolored autoradiograms at respective time points (blue = low; red = high). [^3^H]-CP-55,940 binding was decreased following 2 days of binge ethanol treatment, but this effect was normalized in rats treated for 4 days. * *p* < 0.05 versus control at respective time point. Hippocampus (HC); dorsal dentate gyrus (dDG); Cornu Ammonis 3 (CA3); Cornu Ammonis 1 (CA1); ventral dentate gyrus (vDG); entorhinal cortex (eCTX).

To investigate the effect of neurotoxic binge ethanol exposure on the CB1 receptor, [^3^H]-CP-55,940 binding was examined in the hippocampus and entorhinal cortex (brain regions most susceptible to binge ethanol neurotoxicity [62,63]) following 1, 2 and 4 days of treatment (Figure 1). In the hippocampus, two-way ANOVA revealed a main effect of ethanol treatment (F_(1, 25)_ = 6.692; *p* < 0.05) and planned comparisons revealed a significant decrease in [^3^H]-CP-55,940 binding following two days of binge ethanol treatment versus control diet. In the entorhinal cortex, two-way ANOVA revealed a main effect of duration (F_(2, 24)_ = 4.045; *p* < 0.05) and a diet by duration interaction [F_(2, 24)_ = 4.041; *p* < 0.05]. Planned comparisons revealed a decrease in [^3^H]-CP-55,940 binding following two days of binge ethanol treatment versus control diet (*p* < 0.05). A subsequent analysis examined [^3^H]-CP-55,940 binding in subregions of the hippocampus, including the dorsal dentate gyrus, ventral dentate gyrus, CA3 and CA1 (Figure 1). Although [^3^H]-CP-55,940 binding appeared lower in each layer examined, a significant main effect of diet was only observed in the dorsal dentate gyrus (F_(1, 25)_ = 11.82; *p* < 0.05). Planned comparisons revealed a decrease in [^3^H]-CP-55,940 binding in the dorsal dentate gyrus following two days of binge ethanol treatment (*p* < 0.05).

**Figure 2:**
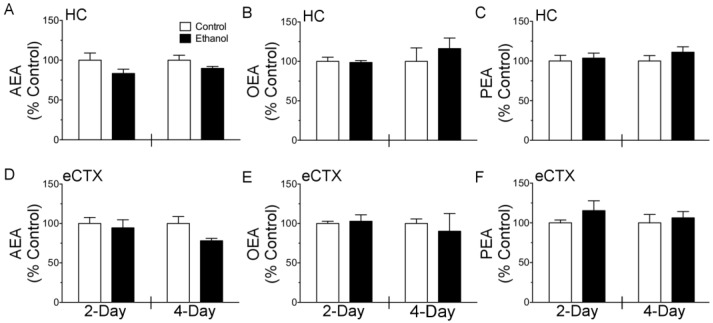
Quantification of anandamide (AEA), oleoylethanolamine (OEA), and palmitoylethanolamine (PEA) bulk tissue content in the hippocampus and entorhinal cortex following binge ethanol treatment. Rats (*n* = 5–6/group) were administered ethanol or an isocaloric control diet for either 2 or 4 days and N-acylethanolamine tissue content was measured in hippocampus and entorhinal cortex by LC-MS and expressed as percent of time point matched controls. (**A**–**C**) AEA, OEA, and PEA tissue content in the hippocampus. (**D**–**F**) AEA, OEA, and PEA tissue content in the entorhinal cortex. Hippocampus (HC); entorhinal cortex (eCTX).

The effect of binge ethanol exposure on three prototypical NAE’s, AEA, OEA, and PEA was measured in the hippocampus and entorhinal cortex following two and four days of treatment (Figure 2). In the hippocampus, two-way ANOVA revealed a main effect of diet for AEA (F_(1, 20)_ = 5.559; *p* < 0.05), however planned comparisons of ethanol vs control were not significant at either two or four days. Binge ethanol treatment did not alter tissue content of AEA in the entorhinal cortex, or PEA and OEA in either brain region examined.

### 3.2. URB597 Administration Failed to Attenuate Binge Ethanol-Induced Degeneration of the Corticolimbic Pathway

**Figure 3:**
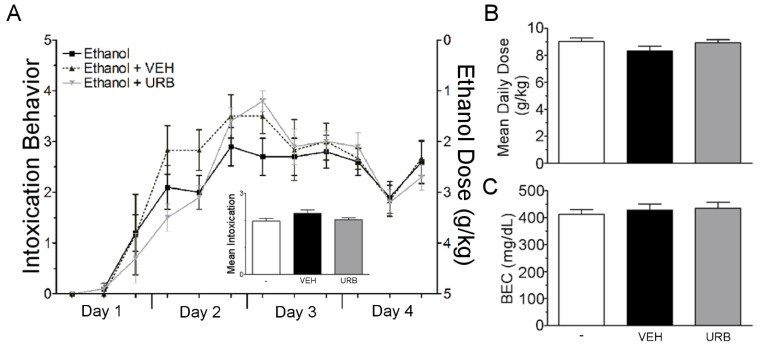
Binge parameters for neuroprotection study. Rats received ethanol according to a modified Majchrowicz binge model and were co-administered nothing (*n* = 10), vehicle (*n* = 6) or URB597 (0.3 mg/kg, *n* = 10) every 12 h starting after the third dosing of ethanol or control diet. (**A**) Ethanol intoxication was assessed prior to each ethanol dosing according to the behavioral intoxication scale described in the methods. Vehicle and URB579 had no effect the intoxicating effects of ethanol over the 4 days of binge treatment (left axis) and the mean intoxication for each group was similar (inset). Ethanol dosing across binge treatment was also similar between groups (right axis). (**B**,**C**) Mean daily dose and blood ethanol concentrations were not different between drug treatment groups Vehicle (VEH); URB597 (URB597); blood ethanol concentration (BEC).

The second objective of these studies was to determine whether engaging NAE signaling affords neuroprotection in a model of binge ethanol exposure. To this end, the FAAH inhibitor, URB597 (0.3 mg/kg), vehicle, or no injection was administered to rats that received concomitant ethanol or control diet according to the four-day binge model. No significant differences were seen in intoxication behavior, ethanol dose, or BEC across treatment groups, ruling out confounding effects of vehicle or drug treatment on neuroprotective measures (Figure 3). 

**Figure 4:**
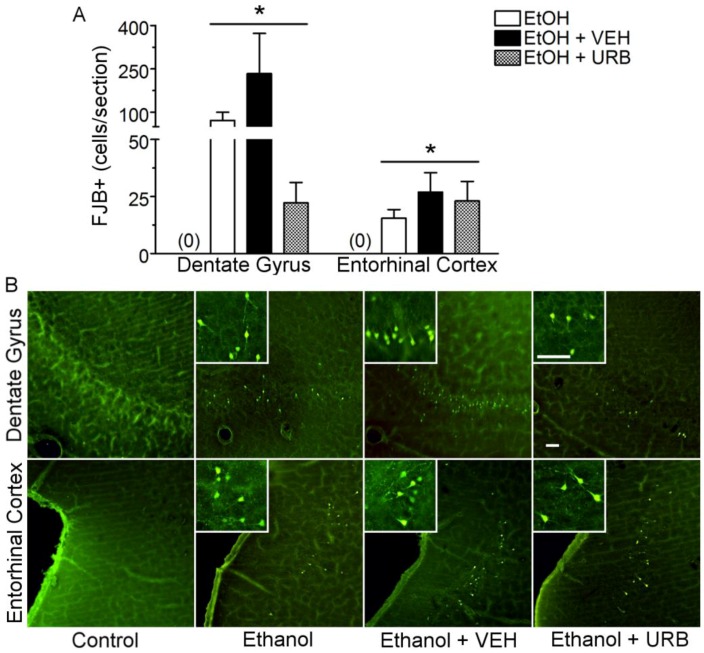
Fatty acid amide hydrolase inhibition by URB597 failed to attenuated binge ethanol induced neurodegeneration of cortico-limbic circuitry. Rats received ethanol or control diet according to a modified Majchrowicz binge model and were co-administered nothing (*n* = 5 for control diet, *n* = 10 for ethanol diet), vehicle (*n* = 5 for control diet, *n* = 6 for ethanol diet) or URB597 (0.3 mg/kg, *n* = 5 for control diet, *n* = 10 for ethanol diet) every 12 h starting after the third dose of ethanol or control diet. Following binge ethanol treatment, neurotoxicity was assessed in the ventral dentate gyrus of the hippocampus and in the entorhinal cortex by FluoroJade B (FJB) staining. (**A**) Quantification of FJB+ neurons in the dentate gyrus and entorhinal cortex. No FJB+ cells were observed in controls as indicated by (0). URB597 failed to significantly attenuate neurotoxicity in either brain region of interest. (**B**) Representative images of FJB+ staining. Note the neuronal morphology of FJB+ cells. Scale bar = 50 µm. * *p* < 0.05 as compared to FJB+ cell counts in respective brain regions of control treated rats. Ethanol (EtOH); vehicle (VEH); URB597 (URB).

Consistent with other reports using the modified Majchrowicz binge model to assess neurodegeneration, FJB+ cells were observed in the granular cell layer of the ventral dentate gyrus and in layers II and III of the entorhinal cortex ([61,62,63], Figure 4). These cells displayed the typical morphology of neurons in these brain regions, including pyramidal-shaped cell bodies and/or a proximal dendritic arbor. Additionally, these cells showed evidence of necrosis, including shrunken cell bodies, which is consistent with previous characterization of binge ethanol induced necrotic cell death [55]. In the ventral dentate gyrus, controls typically had <1 cell/section and were statistically similar, therefore control groups were collapsed before analysis. One-way ANOVA of log-transformed cell counts, revealed an effect of treatment (F_(3, 47)_ = 484.1, *p* < 0.001). Post-hoc analysis showed that binge ethanol treatment resulted in a significant increase in FJB+ labeling in the ventral dentate gyrus (*p* < 0.001) of ethanol-exposed rats. Although URB597 administration appeared to reduce FJB+ cell counts, this group was not significantly different to either the ethanol only or ethanol + vehicle groups due to high variance in FJB+ cell counts in the ventral dentate gyrus. In the entorhinal cortex, controls also displayed <1 cell/section and were therefore collapsed across drug treatment before analysis by one-way ANOVA, which revealed an effect of treatment (F_(3, 37)_ = 1501, *p* < 0.001). Post-hoc analysis showed that binge ethanol exposure resulted in a significant increase in FJB+ cells in the entorhinal cortex (*p* < 0.001). However, URB597 treatment did not reduce FJB+ cells when compared to the ethanol only or ethanol + vehicle groups. 

As neuroprotection was not observed, statistically, following pharmacological inhibition of FAAH, the next experiment was designed to determine whether binge ethanol treatment interfered with URB597-mediated augmentation of AEA, OEA, and PEA in the hippocampus and entorhinal cortex. Rats were treated with ethanol or control diet for a single day (three doses) and were administered an acute dose of URB597 (0.3 mg/kg) 2 h prior to euthanasia. Ethanol exposure was not different across treatment groups: all intoxication parameters were statistically similar (see Table 2). 

**Figure 5:**
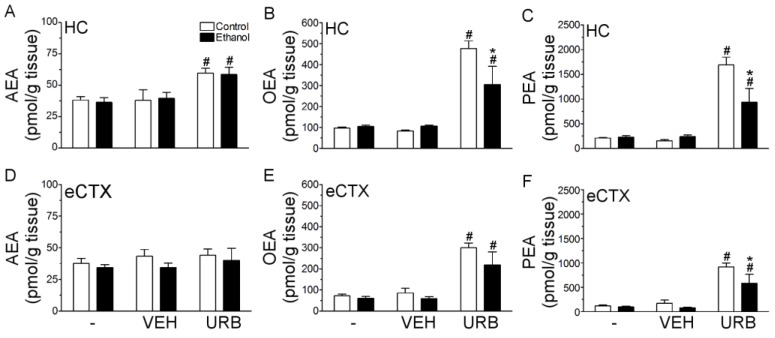
N-acylethanolamine (NAE) augmentation following an acute dose of URB597 is attenuated by binge ethanol exposure. Rats (*n* = 5–6/group) received binge ethanol treatment for 1 day (3 doses) and were then administered nothing, vehicle, or a single dose of URB597 (0.3 mg/kg). Two hours following drug treatment, rats were euthanized and NAE tissue content was assessed in the hippocampus and entorhinal cortex. (**A**–**C**) URB597 augmented anandamide (AEA), oleoylethanolamine (OEA), and palmitoylethanolamine (PEA) in the hippocampus of control and binge ethanol treated animals, however the magnitude of OEA and PEA elevation was blunted in binge ethanol treated animals relative to controls. (**D**–**F**) URB597 augmented OEA and PEA, but not AEA in the entorhinal cortex. In binge ethanol treated animals, URB797 failed to elevate PEA tissue content to the same magnitude as in control animals. # *p* < 0.05 as compared to diet matched VEH treated animals; * *p* < 0.05 when compared control animals treated with URB597. Hippocampus (HC); entorhinal cortex (eCTX); vehicle (VEH); URB597 (URB).

In the hippocampus, two-way ANOVAs revealed a main effect of drug treatment on AEA (F_(2, 30)_ = 10.82; *p* < 0.001), OEA (F_(2, 30)_ = 36.91; *p* < 0.001), and PEA (F_(2, 30)_ = 46.74; *p* < 0.001) tissue content (Figure 5A–C). Additionally, there was a significant diet x drug interaction for OEA (F_(2, 30)_ = 3.781; *p* < 0.05) and PEA (F_(2, 30)_ = 6.381; *p* < 0.01) in the hippocampus. In control rats, post-hoc analyses found that URB597 elevated AEA by 57.6% (*p* < 0.05), OEA by 475.4% (*p* < 0.001) and PEA by 986.6% (*p* < 0.001) when compared to rats treated with vehicle. Although NAE augmentation was much lower in ethanol treated, post-hoc analyses showed that URB597 elevated AEA by 48.1% (*p* < 0.05), OEA by 188.8% (*p* < 0.01), and PEA by 287.2% (*p* < 0.01) relative to ethanol + vehicle treated rats. Importantly, ethanol attenuated URB597-mediated augmentation of OEA by 36.3% (*p* < 0.05) and PEA by 44.7% (*p* < 0.001) in the hippocampus compared to binge ethanol naïve rats.

In the entorhinal cortex (Figure 5D–F), two-way ANOVA revealed a significant effect of drug treatment on the levels of OEA (F_(2, 29)_ = 27.40; *p* < 0.001) and PEA (F_(2, 29)_ = 34.63; *p* < 0.001), but not on AEA (F_(2, 29)_ = 0.60; *p* > 0.05). In control rats, planned comparisons revealed that URB597 administration elevated OEA by 250.3% (*p* < 0.001) and PEA by 435.0% (*p* < 0.001) as compared to rats that received vehicle. This effect was also evident in binge ethanol treated rats as URB597 elevated OEA by 268.6% (*p* < 0.001) and PEA by 637.7% (*p* < 0.001) when compared to rats treated with vehicle. There was also a main effect of diet on PEA levels in the entorhinal cortex (F_(1, 29)_ = 4.344; *p* < 0.05). Planned comparison showed that PEA content following URB597 administration was 36.7% lower in binge ethanol treated rats compared to ethanol naïve rats (*p* < 0.05). 

**Figure 6:**
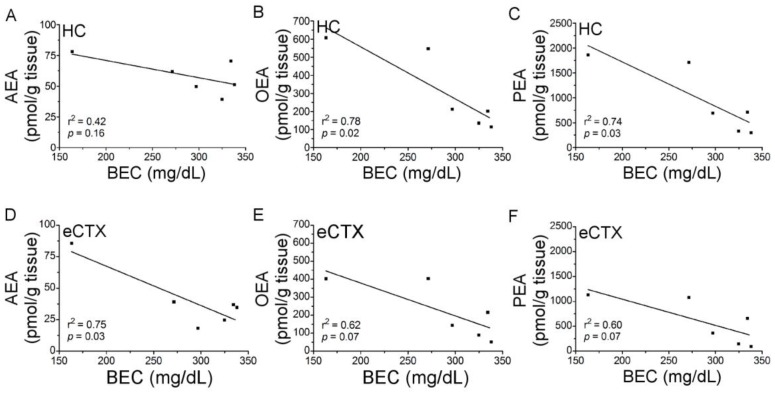
Correlation analysis of blood ethanol concentrations (BECs) and N-acylethanolamine (NAE) tissue content following co-administration of binge ethanol and URB597. Rats (*n* = 5–6/group) received binge ethanol treatment of one day (three doses) and a single dose of URB597 (0.3 mg/kg). Two hours following drug treatment, rats were euthanized and NAE tissue content and BECs were assessed. (**A**–**C**) Hippocampal tissue content of AEA, OEA, and PEA are negatively correlated with BEC. (**D**–**F**) Entorhinal cortex tissue content of AEA, OEA, and PEA, are negatively correlated with BEC. Hippocampus (HC); entorhinal cortex (eCTX).

In order to further probe the relationship between ethanol treatment and URB597 augmentation of the NAEs, Pearson correlations were performed on BECs vs. NAE content (Figure 6). In the hippocampus, OEA (r^2^ = 0.78; *p* < 0.05) and PEA (r^2^ = 0.74; *p* < 0.05) were significantly correlated with BECs. In the entorhinal cortex, AEA content was significantly correlated with BECs (r^2^ = 0.75; *p* < 0.05), while correlations for OEA (r^2^ = 0.62) and PEA (r^2^ = 0.60) vs. BECs only trended towards significance (*p* = 0.07). 

## 4. Discussion

The aim of the current study was to characterize the effect of neurotoxic binge alcohol treatment on components of the eCB system and to determine if enhancement of eCB/NAE tissue content is an efficacious strategy for attenuating binge alcohol-induced neurodegeneration. In the current study, [^3^H]-CP-55,940 binding was observed in cortico-limbic circuitry, however this effect was transient, which was only evident following two days of binge alcohol treatment. Furthermore, binge alcohol exposure did not change the tissue content of AEA, OEA, or PEA in the hippocampus or entorhinal cortex. These data suggest that CB1 receptor expression and/or function, and NAE metabolism is resilient to neurotoxic patterns of alcohol exposure and may be permissive to pharmacological modulation for attenuating alcohol-induced neuronal cell death. To that end, the neuroprotective effects of FAAH inhibition were evaluated. Repeated administration of URB597 failed to significantly decrease FJB+ cells in either the entorhinal cortex or ventral dentate gyrus of the hippocampus, suggesting that FAAH inhibition does not preserve cortico-limbic circuitry following binge alcohol exposure. However, a follow up “target engagement” experiment found that augmentation of NAE tissue content following an acute dose of URB597 was less robust in alcohol-intoxicated animals.

CB1 receptors have emerged as a promising therapeutic target for the treatment of many neurodegenerative disorders [41], and may also be a viable target for mitigating the neurotoxic effects of excessive alcohol consumption. However, previous studies have reported alcohol-induced downregulation of CB1 in many regions of the CNS [28,71,72,73], which may diminish the efficacy of neuroprotective therapies targeting this receptor and/or lead to increased vulnerability to the neurotoxic effects of alcohol. Therefore, a primary aim of this study was to determine the impact of neurotoxic binge alcohol exposure on CB1 receptors in the hippocampus and entorhinal cortex, regions susceptible to alcohol-induced neurodegeneration. [^3^H]-CP-55,940 binding was decreased following two days of binge ethanol treatment, however, this effect was only transient as [^3^H]-CP-55,940 binding following the full four-day binge paradigm was similar to controls. The transient nature of [^3^H]-CP-55,940 binding was surprising because in other models using similar durations of alcohol exposure, with comparable BECs, CB1 receptor down regulation normalizes subsequent to withdrawal [71,74]. However, the effects of alcohol on the eCB system are dependent on many experimental variables, including model organism [27,28,29], and the current study was performed in male Sprague Dawley rats, while the other reports were performed in male Swiss Webster and C57BL6/J mice [71,74]. The mechanism of decreased [^3^H]-CP-55,940 binding was not examined in the current study, but others have suggested receptor desensitization as a possible mechanism [71]. Although we did not see concurrent increases in AEA following two days of binge treatment, our results do not rule out CB1 receptor desensitization following persistent 2-arachidonoylglycerol (2-AG) signaling by alcohol exposure. 2-AG tissue levels were not included in the current study because we had previously developed a LC-MS method [68] to monitor levels of the N-acylethanolamines, AEA, PEA, and OEA, which turned out to be incompatible with simultaneous measurement of 2-AG. Nevertheless, the current results suggest that CB1 receptors are relatively resistant to binge alcohol exposure and may be a viable target for neuroprotective therapies.

The ECs and related NAEs have a variety of neuroprotective properties and are responsible for limiting neuronal damage following CNS perturbation [75]. In particular, the NAEs, including AEA, OEA, and PEA, are synthesized and accumulate in damaged tissue, an effect that has been observed over a range of CNS injury models [31,32,33,34,35,36]. These observations have led to the hypotheses that the NAEs and the signaling cascades activated by these lipids represent an endogenous neuroprotective system. Although EC and NAE engagement has been observed in a variety of models of CNS injury and/or neurodegeneration, this response has not been investigated in the context of alcohol-induced neurodegeneration. In the current study, three prototypical NAEs, AEA, OEA, and PEA were quantified in the entorhinal cortex and hippocampus following binge alcohol treatment. In contrast to other models of neurodegeneration, elevation in NAE tissue content was not observed following binge alcohol induced neurodegeneration. The four-day binge model that was used in the current study results in necrotic cell death in cortico-limbic circuitry, which is most prominent in the entorhinal cortex and ventral dentate gyrus of the hippocampus [55,61,62]. Therefore, it was unexpected that NAEs were unchanged in either of these brain regions, especially following four-days of binge exposure, a time point that is associated with maximal neuronal damage [62,63]. 

The lack of NAE accumulation may be related to the magnitude and/or the nature of neurodegeneration that occurs during binge alcohol exposure. For example, when NAE accumulation was compared across three models of experimental neurodegeneration, robust NAE accumulation only occurred following N-methyl-D-aspartate (NMDA) mediated excitotoxicity. Only moderate to marginal NAE elevations were observed following head injury and NMDA receptor antagonism, respectively [34]. Relative to either of the latter insults, the magnitude of cortico-limbic neurodegeneration observed following binge alcohol exposure is minor and therefore may not stimulate substantial NAE synthesis. Further, if binge alcohol only results in a minor and localized accumulation of NAEs, then the current method for quantification may not detect such subtle changes. For example, binge alcohol induced neuronal damage occurs in layers II and III of the entorhinal cortex and within the granular cell layer of the ventral dentate gyrus of the hippocampus; however, NAEs were quantified using the entire entorhinal cortex and hippocampus. Therefore, potential local elevations in NAE content could be diluted during quantification. NAE biosynthesis, which has been shown to be stimulated by rises in intracellular Ca^2+^, may be less sensitive to necrotic cell death following four-days of binge alcohol exposure since neurodegeneration in this model appears to be independent of NMDA receptors and other voltage-gated Ca^2+^ channels [76,77]. In all likelihood, a relatively mild injury severity and a potential lack of involvement from Ca^2+^ permeable channels, contributes to the absence of NAE accumulation during binge alcohol induced neurodegeneration.

Repeated administration of URB597 over the course of binge ethanol treatment did not result in significant protection of cortico-limbic circuitry, as assessed by FJB+ cell counts. We only tested a single dose of URB597 at 0.3 mg/kg because an initial characterization of URB597 by Fegley et al. [69], found that this dose maximally inhibited FAAH in rat brain for up to 12 h. Therefore, with the dosing protocol used in the current study, FAAH was expected to be maximally inhibited over the course of treatment. However, since repeated administration of URB597 failed to afford neuroprotection, we tested the effect of URB597 on the accumulation of AEA, OEA, and PEA in the hippocampus and entorhinal cortex to ensure “target engagement” following simultaneous binge alcohol exposure and URB597 treatment. In alcohol naive rats, URB597 significantly elevated AEA, OEA, and PEA in the hippocampus, and OEA and PEA in the entorhinal cortex. In binge alcohol treated rats, URB597 resulted in similar patterns of AEA, OEA, and PEA accumulation, but at significantly reduced levels. Furthermore, correlation analysis suggested that BECs may be an important factor associated with diminished URB597-mediated NAE elevation. In fact, two rats with BECs approaching 350 mg/dL failed to have detectable elevations in NAE content following URB597 administration. With this in mind, BECs from rats in the neuroprotection study averaged over 400 mg/dL, therefore, URB597-mediated NAE elevation may have been severely diminished over the course of binge treatment. Thus, the reduced effect of URB597 in binge alcohol treated rats may explain the lack of neuroprotection. Future studies should examine whether this reduced effect could be overcome by higher doses of URB597. 

Additionally, pharmacodynamic tolerance may develop to URB597, as one study found that an acute dose of URB597 resulted in the augmentation of NAE content in the spinal cord, though, this effect was lost after repeated dosing [78]. Interestingly, this tolerance was associated with reduced N-acyl phosphatidylethanolamine phospholipase D (NAPE-PLD) protein expression. Therefore, adaptations in NAE biosynthetic pathways, following repeated dosing, could contribute to the lack of neuroprotection that was observed in the current study. Lastly, an important consideration is the effect of alcohol exposure on the metabolism of NAEs. Studies have shown that acute alcohol administration can decrease NAE content [79], while chronic alcohol exposure is associated with decreased FAAH expression and/or activity [71]. Therefore, neuroadaptations in NAE synthesis and degradation in response to alcohol exposure may impact the pharmacodynamics of URB597. 

Another important finding in the current study was that URB597-mediated NAE elevation was brain region and NAE specific. For example, the enhancement of AEA was observed in the hippocampus but not in the entorhinal cortex. Additionally, URB597 had different relative magnitudes of effect as PEA and OEA were elevated substantially, while AEA was affected marginally. This effect is consistent with another study reporting brain region-specific elevations in AEA following URB597 administration [49]. The brain region-specific effects of FAAH inhibition observed in the current study may be attributed to the expression patterns of FAAH in the CNS, as FAAH expression, assessed by immunohistochemistry, is more abundant in the hippocampus when compared to the entorhinal cortex [80]. 

## 5. Conclusions

In conclusion, FAAH inhibition does not appear to afford neuroprotection in the context of binge alcohol neurotoxicity, which may be a result of altered pharmacodynamics of URB597 in the presence of highly intoxicating concentrations of alcohol. Although neuroprotection was not observed in the model used in the current study, FAAH inhibition may be a beneficial treatment for other aspects of alcohol dependence, such as alcohol withdrawal [51]. Additionally, it was our goal to examine the potential neuroprotective effects of FAAH inhibition specifically as inhibition of this enzyme is predicted to not only engage eCB signaling pathways, but also other pathways that are associated with cell survival that are engaged by the NAEs, independent of cannabinoid receptors [81]. However, since this strategy failed, future studies should examine 2-AG, test whether modulation of 2-AG metabolism could afford neuroprotection, and examine other pharmacological agents that engage the eCB system, such as monoacylglycerol lipase inhibitors, which may prove more efficacious in attenuating alcohol induced neurodegeneration. 

## Figures and Tables

**Table 1:** Binge Parameters.

Group	Intoxication Behavior *	Ethanol Dose (g/kg/day)	BEC (mg/dL)
**CB1 Autoradiography**			
1-Day (*n* = 5)	0.5 ± 0.1	13.4 ± 0.4	304.1 ± 19.0
2-Day (*n* = 6)	1.6 ± 0.1	10.3 ± 0.3	403.2 ± 28.0
4-Day (*n* = 6)	2.1 ± 0.2	8.8 ± 0.4	404.9 ± 24.4
**NAE quantification**			
2-Day (*n* = 6)	1.4 ± 0.2	10.8 ± 0.6	443.8 ± 10.5
4-Day (*n* = 7)	2.3 ± 0.1	8.1 ± 0.3	420.5 ± 24.1

* mean intoxication behavior (0–5 scale, see text for details); NAE, N-acylethanolamide; BEC, blood ethanol concentration.

**Table 2:** Binge Parameters.

Group	Intoxication Behavior *	Ethanol Dose (g/kg/day)	BEC (mg/dL)
No Injection (*n* = 5)	0.3 ± 0.1	13.8 ± 0.4	331.8 ± 27.8
Vehicle (*n* = 6)	0.1 ± 0.1	14.7 ± 0.2	304.3 ± 18.4
URB597 (*n* = 6)	0.2 ± 0.2	14.3 ± 0.2	291.4 ± 28.4

* mean intoxication behavior (0–5 scale, see text for details); blood ethanol concentration (BEC).
